# Western-type diet influences mortality from necrotising pancreatitis and demonstrates a central role for butyrate

**DOI:** 10.1136/gutjnl-2019-320430

**Published:** 2020-09-01

**Authors:** Fons F van den Berg, Demi van Dalen, Sanjiv K Hyoju, Hjalmar C van Santvoort, Marc G Besselink, Willem Joost Wiersinga, Olga Zaborina, Marja A Boermeester, John Alverdy

**Affiliations:** 1 Department of Surgery, Amsterdam UMC, University of Amsterdam, Amsterdam Gastroenterology Endocrinology Metabolism, Amsterdam, The Netherlands; 2 Department of Surgery, Radboudumc, Nijmegen, The Netherlands; 3 Department of Surgery, University of Chicago, Pritzker School of Medicine, Chicago, Illinois, USA; 4 Department of Surgery, Regional Academic Cancer Center Utrecht, UMC Utrecht Cancer Center, Utrecht, The Netherlands; 5 Department of Surgery, Sint Antonius Hospital, Nieuwegein, The Netherlands; 6 Center for Experimental and Molecular Medicine, Department of Medicine, Amsterdam UMC, University of Amsterdam, Amsterdam, The Netherlands

**Keywords:** acute pancreatitis, bacterial infection, diet, butyrate, antibiotics

## Abstract

**Objective:**

The gut microbiota are the main source of infections in necrotising pancreatitis. We investigated the effect of disruption of the intestinal microbiota by a Western-type diet on mortality and bacterial dissemination in necrotising pancreatitis and its reversal by butyrate supplementation.

**Design:**

C57BL/6 mice were fed either standard chow or a Western-type diet for 4 weeks and were then subjected to taurocholate-induced necrotising pancreatitis. Blood and pancreas were collected for bacteriology and immune analysis. The cecum microbiota composition of mice was analysed using 16S rRNA gene amplicon sequencing and cecal content metabolites were analysed by targeted (ie, butyrate) and untargeted metabolomics. Prevention of necrotising pancreatitis in this model was compared between faecal microbiota transplantation (FMT) from healthy mice, antibiotic decontamination against Gram-negative bacteria and oral or systemic butyrate administration. Additionally, the faecal microbiota of patients with pancreatitis and healthy subjects were analysed.

**Results:**

Mortality, systemic inflammation and bacterial dissemination were increased in mice fed Western diet and their gut microbiota were characterised by a loss of diversity, a bloom of *Escherichia coli* and an altered metabolic profile with butyrate depletion. While antibiotic decontamination decreased mortality, Gram-positive dissemination was increased. Both oral and systemic butyrate supplementation decreased mortality, bacterial dissemination, and reversed the microbiota alterations. Paradoxically, mortality and bacterial dissemination were increased with FMT administration. Finally, patients with acute pancreatitis demonstrated an increase in Proteobacteria and a decrease of butyrate producers compared with healthy subjects.

**Conclusion:**

Butyrate depletion and its repletion appear to play a central role in disease progression towards necrotising pancreatitis.

**Significance of this study**

**What is already known about this subject?**

- Gut microbiota is affected in patients with acute pancreatitis, as compared with healthy individuals.
- Microbes that cause secondary infection of necrotising pancreatitis originate from the gastrointestinal tract.
- Increased intestinal permeability and altered gut microbiota following acute necrotising pancreatitis are associated with infectious complications.
- Obesity is a risk factor for adverse outcome by increasing (peri) pancreatic infections and inflammation, although the mechanism is unclear.

**What are the new findings?**

- Western diet feeding aggravated experimental acute necrotising pancreatitis by increasing bacterial dissemination and altered the intestinal metabolic profile with depletion of short-chain fatty acids.
- *Escherichia coli* dominated among the microbiota of Western-diet fed pancreatitis mice, and selective Gram-negative decontamination attenuated Gram-negative bacteria but conversely increased Gram-positive bacterial dissemination.
- In a mouse model of acute necrotising pancreatitis, faecal microbiota transplantation increased bacterial dissemination and mortality. In contrast, butyrate supplementation, both prepancreatitis and postpancreatitis inductions, attenuated disease progression in mice.

**Significance of this study**

**How might it impact on clinical practice in the foreseeable future?**

- Our study indicates that prophylactic butyrate administration in patients with acute pancreatitis is a potential strategy for preventing disease progression in this potentially lethal disease.

## Introduction

Acute pancreatitis (AP) has a global incidence of 33.74 cases and 1.6 deaths per 100 000 person-years.^1^ Severe infections are often debilitating and a major cause of mortality in these patients. Most often, blood, sputum and pancreatic tissue samples are culture positive for bacteria that normally reside at low abundance in the gastrointestinal tract, such as *Escherichia coli, Klebsiella* spp and *Enterococcus* spp. Bacteraemia is a risk factor for and often precedes the occurrence of infected necrotising pancreatitis.^2^ Previous studies have shown that increased permeability of the gut barrier and altered intestinal microbiota are associated with infectious complications.^3 4^ This supports the idea that infected necrotising pancreatitis can be caused by gut bacteria that translocate to (peri) pancreatic collections.

In this study, we hypothesised that an altered composition and functional capacity of the intestinal microbiota can be identified that precedes and predicts the onset of acute necrotising pancreatitis (ANP) and is exaggerated by the intake of a Western-type diet (WD). Therefore, the aim of this study was to assess the role of the intestinal microbiota on the development of ANP in mice fed a WD. Results revealed a direct link between depletion of the commensal microbiota and lethal infections in AP which can be prevented by butyrate administration and to a lesser degree by antibiotic decontamination but not by faecal microbiota transplant (FMT). Evaluation of the role of the microbiota in ANP in the context of a Western diet may inform novel preventative therapy.

## Materials and methods

### Mice

Six-week to eight-week-old C57BL/6 mice were purchased (Charles River Laboratories International, Wilmington, Massachusetts, USA) and housed under standard conditions (12 hours dark/light cycle) for at least 72 hours before any study procedures were performed. The animals had ad libitum access to tap water and either standard chow diet (SD) or a WD that contained 60% polyunsaturated fatty acids (S3282, Bio-Serv, Flemington, New Jersey, USA) and no soluble fibres, as previously described.^5^ The animals were sacrificed by CO suffocation at either 24 hours or 72 hours following surgery, or when moribund. Blood was drawn via cardiac puncture, followed by aseptic removal of pancreas and cecum tissue and contents. Aliquots were stored at −80°C until analysis.

### Mouse model of biliary, necrotising pancreatitis

Necrotising pancreatitis was induced by infusion of the pancreatic duct with taurocholate acid (TA), as previously described.^6^ A detailed description of procedures is provided in the online supplementary methods.

10.1136/gutjnl-2019-320430.supp1 Supplementary data

### Treatments

For oral butyrate supplementation, butyric acid (100 mM, Sigma-Aldrich, Saint-Louis, Missouri, USA) was added to the drinking water (changed weekly) for 4 weeks prior to the procedure. For systemic butyrate or histone deacetylase (HDAC) inhibitor treatment, animals were injected intraperitoneally with 500 uL sodium butyrate (40 mM) or trichostatin A (TSA 133 μg/mL) in sterile saline at 0, 7 and 14 hours postoperatively. Control groups were injected with 500 uL sterile saline.

After 23 days of WD feeding, selective Gram-negative gut depletion (GNGD) was carried out by oral gavage of neomycin (100 mg/kg body weight) and polymyxin B (25 mg/kg body weight) dissolved in sterile saline two times per day for 5 days.^7^ The control group received non-supplemented, normal drinking water.

For the faecal transplants, fresh faecal pellets were collected from 6-week to 8-week-old C57BL/6 mice in saline with a final concentration of 50 mg faeces/mL. Pooled samples were centrifuged (100×g for 2 min) to pellet large particles and the supernatant used for FMT treatment. For sterile faecal filtrate (SFF), the supernatants were collected and passed through 30, 0.45 and 0.22 µm filters (Millipore). A total of 200 µL of FMT or SFF was administered per mice via oral gavage, starting approximately 1 hour after the procedure and then every 24 hours until sacrifice.

### Bacterial phenotype microarray analysis, targeted (short-chain fatty acids) and untargeted metabolomics

Following sacrifice, cecum content was collected, snap frozen and stored at −80°C until analysis. For the bacterial phenotype microarray analysis, an aliquot was stored in 10% glycerol. The analysis was performed on GEN-III Biolog plates as previously described.^5^ Targeted gas chromatography-mass spectrometry (GC-MS) analysis was performed to measure short-chain fatty acids (SCFAs). Untargeted GC-MS analysis was annotated with the Fiehn GC-MS metabolites RTL library. Detailed procedures for the phenotype microarray and metabolomics analysis are provided in the online supplementary file.

### 16S rRNA gene amplicon sequencing

Microbial DNA was extracted from cecum tissues and content using the MagAttract PowerMicrobiome DNA/RNA KF kit. Amplicons of the V4 region of the 16S rRNA gene were constructed using 515F/806R primer pair, according to the Earth Microbiome Project protocols (http://www.earthmicrobiome.org/emp-standard-protocols/16s/). Amplicon sequencing was done on a MiSeq platform (Illumina, San Diego, California, USA) at the Argonne Sequencing Facility, generating 150 bp pair-end reads.^8 9^ Forward and reverse reads were merged using USEARCH V.3.^10^ Amplicon Sequence Variants (ASVs) were inferred for using UNOISE V.3 and reads were than mapped against the ASV set to determine the abundances. Taxonomy was assigned using the Ribosomal Database Project (RDP) classifier^11^ and SILVA^12^ 16S ribosomal database V.132. A panel of butyrate producers based on genus taxonomy was constructed based on butyrate-producing taxa (online supplementary table 1).^13^

10.1136/gutjnl-2019-320430.supp2 Supplementary data

### Patients

Thirty-five Dutch patients with predicted severe AP (SAP) who participated in the “Probiotic prophylaxis in predicted severe acute pancreatitis” (PROPATRIA) trial with available stool samples were included.^14^ Patients who participated in this trial received either probiotics or placebo following randomisation. Stool samples were collected within 72 hours of admission, before randomisation and administration of the study products. Severity was defined as SAP (moderate severe or SAP) or mild AP (MAP) according to the revised Atlanta criteria. Additionally, 15 healthy, non-smoking, Caucasian male volunteers who were part of the MISSION-2 project (NCT02127749), with no previous exposure to antibiotics for the last year were included as control group.^15 16^ Stool samples were collected and immediately stored at −20°C until further analysis. Procedures for DNA isolation and 16S rRNA gene amplicon sequencing of human stool samples are provided in the online supplementary methods.

### Statistical analysis

The data were analysed using GraphPad Prism V.8 (Graphpad Software, San Diego, California, USA) or R (R Core Team, https://www.R-project.org/). Unless otherwise stated, results were expressed as mean±SD. Non-parametric Mann-Whitney or Student’s t-test was used to test statistical significance, depending on the distribution of normality. The ‘DESeq’ function ‘DSEeq V.2’ package was used for significance of relative abundance of metabolites for the univariate metabolomics analysis.^17^ Analysis of variance (ANOVA) was used to test significant differences between multiple groups. Permutation Multivariate ANOVA (PERMANOVA) was used for group-wise comparisons of beta-diversity measures using ‘adonis’ from the ‘vegan’ package.^18^ Log-rank test was used for significance testing in Kaplan-Meyer curves (Prism V.8). A significance test based on cross (model) validation with 999 permutations was used to test significant differences between two groups for the sparse partial-least squares discriminant analysis (sPLS-DA) analysis, using the function ‘MWA.test’ of the ‘RVAideMemoire’ package.^19^

## Results

### Infusion of TA into the pancreatic duct induces ANP and mortality in WD-fed mice

**Figure 1:**
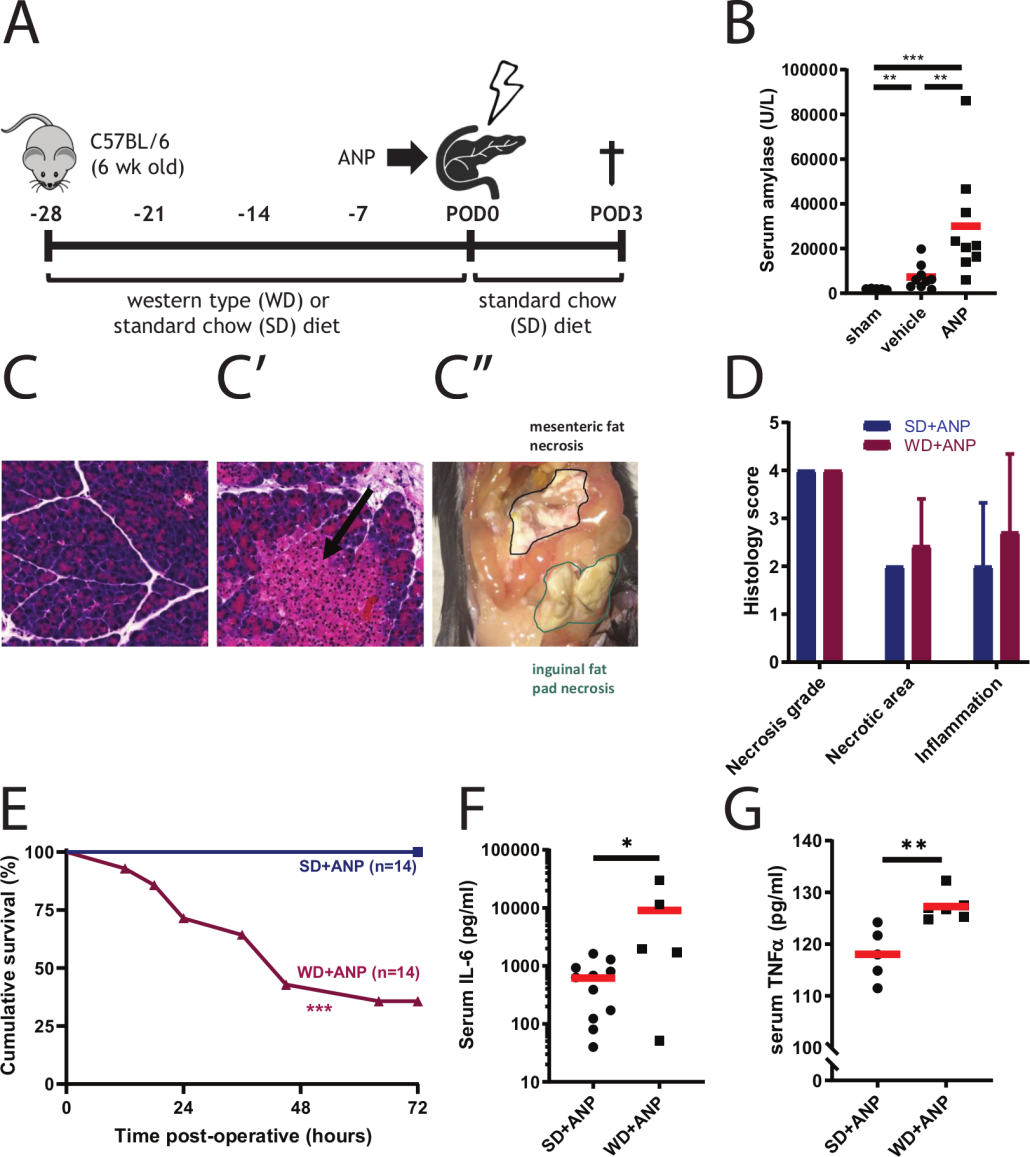
Western diet increases mortality and inflammation in mice with ANP. (A) Mouse model of acute necrotising pancreatitis. (B) Serum amylase levels are significantly increased in mice administrated taurocholate in the pancreatic duct (ANP, n=9) compared with vehicle controls (saline, n=9) (p=0.001) or sham-operated mice (n=5) (p<0.001, Mann-Whitney test). (C–C′′) Microscopic (C, C′) and macroscopic (C′′) histological changes of pancreatic tissue of mice administered vehicle (C) or taurocholate (ANP, C′). Parenchymal necrosis (C′, arrow) and extrapancreatic fat necrosis in mice treated with taurocholate (C′′). (D) Histology score adapted from Schmidt’s criteria (p>0.05 by Mann-Whitney test). (E) Kaplan-Meijer curve of groups of mice that received a SD or WD diet prior to induction of necrotising pancreatitis shows increased mortality in the latter group (p<0.001 by log-rank test). (F and G) Systemic inflammation at 24 hours after taurocholate administration (ANP) measured by serum IL-6 (F) and serum TNFα (G). (F) n=5–10, p=0.036 by unpaired t-test). (G) n=5–6 per group, p=0.004 by unpaired t-test). *p<0.05; **p<0.01; ***p<0.001. ANP, acute necrotising pancreatitis; SD, standard chow diet; WD, Western-type diet.

ANP was induced by retrograde infusion of the pancreatic duct with TA in mice (figure 1A). Necrotising pancreatitis developed in both the SD pancreatitis (SD+ANP) and WD pancreatitis (WD+ANP) groups, which was confirmed by elevated serum amylase (figure 1B). Macroscopic extrapancreatic fat necrosis and microscopic focal pancreatic necrosis were observed in both groups (figure 1C). Histology score accounting for necrosis grade, necrotic area and inflammation as described previously^6^ were comparable among SD+ANP and WD+ANP mice (figure 1D). Four weeks of WD feeding by itself resulted in the increase of body weight (see online supplementary figure 1A) and slightly elevated serum endotoxin level (see online supplementary figure 1B). ANP induction led to a significant increase of endotoxin in both SD-fed and WD-fed mice (see online supplementary figure 1C). However, 64% mortality (9 out of 14) was observed in WD pancreatitis mice, where no deaths were observed in the SD pancreatitis group (figure 1E). This correlated with increased systemic inflammation in the WD+ANP group compared with the SD+ANP group measured at 24 hours post-operatively (figure 1F, G).

10.1136/gutjnl-2019-320430.supp3 Supplementary data

### Culture results demonstrate significant systemic infection in WD+ANP mice

**Figure 2:**
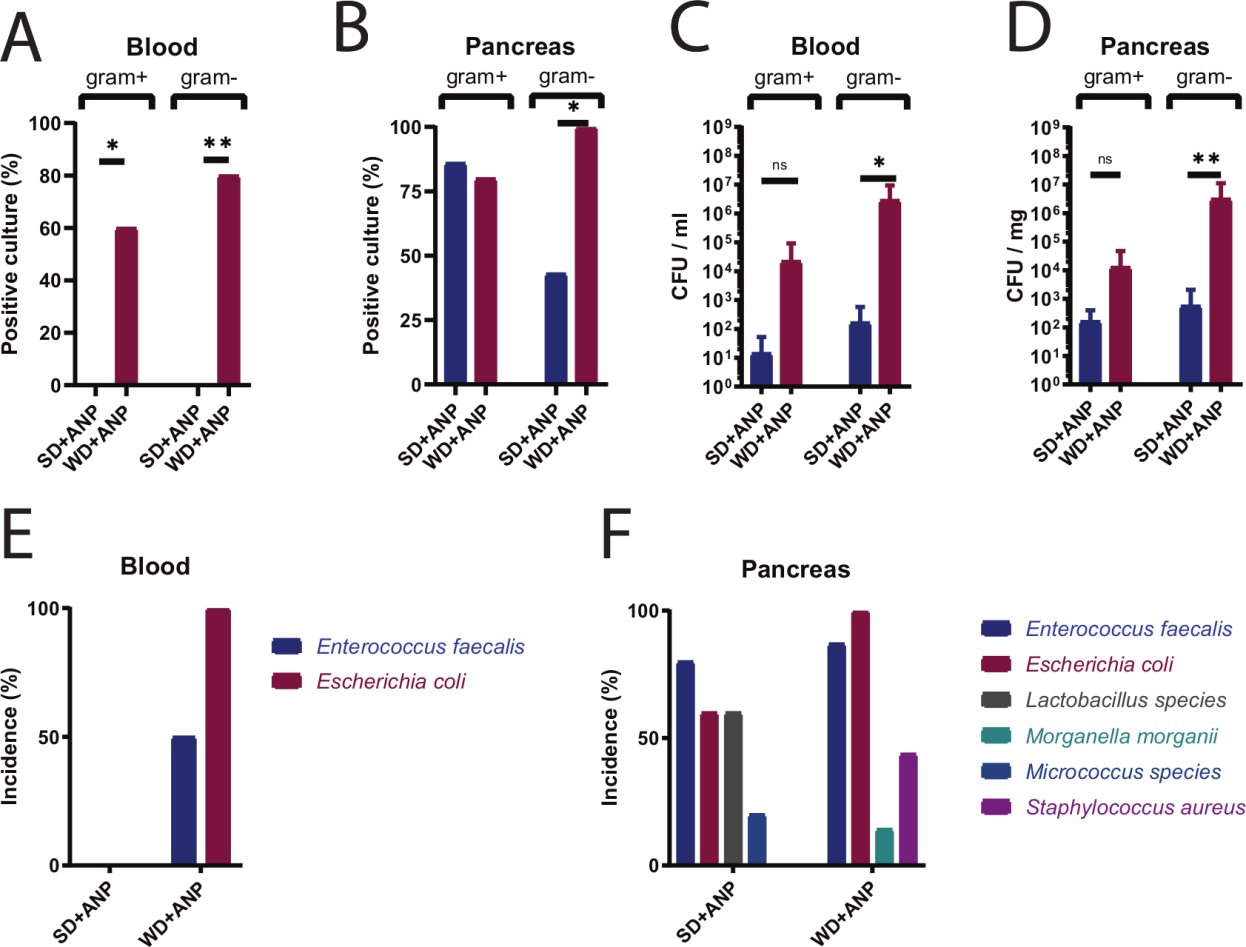
Culture analysis of blood and pancreas in SD-fed and WD-fed ANP mice. Frequency of culture positivity in blood (A) and pancreas tissue (B). MacConkey media were used for Gram-negative and colistin/nalidixic acid (CNA) 5% sheep blood agar for Gram-positive bacteria, n=7–10 per group, p=0.002 blood Gram-positive, p=0.035 blood Gram-negative, p=0.015 for pancreas Gram-negative. Bacterial density (CFU) in blood (C) and pancreas (D) in mice at the lethal endpoint (moribund or 72 hours postoperatively). Significant differences were found for Gram-negative bacteria between WD-fed and SD-fed ANP mice (n=7–10 per group, p=0.013 in blood; p=0.003 in pancreas tissue by Mann-Whitney test). Incidence of identified cultured bacterial species in blood (E) and pancreatic tissue (F). *p<0.05; **p<0.01; ns, not statistically significant. ANP, acute necrotising pancreatitis; CFU, colony forming units; SD, standard chow diet; WD, Western-type diet.

Blood and homogenised pancreas tissues were cultured on specific Gram-negative (MacConkey) and Gram-positive (colistin/nalidixic acid 5% sheep blood) plates. There was a significant increase in the frequency of positive blood cultures in WD+ANP versus SD+ANP groups for both Gram-negative (p=0.002, Fisher exact test) and Gram-positive (p=0.035) bacteria (figure 2A). A significant increase in the frequency of positive pancreatic tissue cultures was observed for Gram-negative bacteria only (p=0.015) (figure 2B). The abundance (colony forming units (CFU)/mL blood or CFU/mg pancreatic tissue) of Gram-negative bacteria was significantly higher in WD pancreatitis (figure 2C, D).

The identification of cultured colonies revealed *Enterococcus faecalis* and *E. coli* as the most predominant Gram-positive and Gram-negative bacteria, respectively (figure 2E, F). *E. faecalis* was detected at high frequency in the blood and pancreatic tissue of both WD-fed and SD-fed mice; however, *E. coli* was detected at high frequency only in WD-fed mice (figure 2E, F). Notable was the presence of *Lactobacillus* sp in pancreatic tissue of SD-fed but not WD-fed mice (figure 2F).

### 16S rRNA gene amplicon analysis of cecal microbiota reveals a bloom of *Escherichia/Shigella* in WD+ANP mice

**Figure 3:**
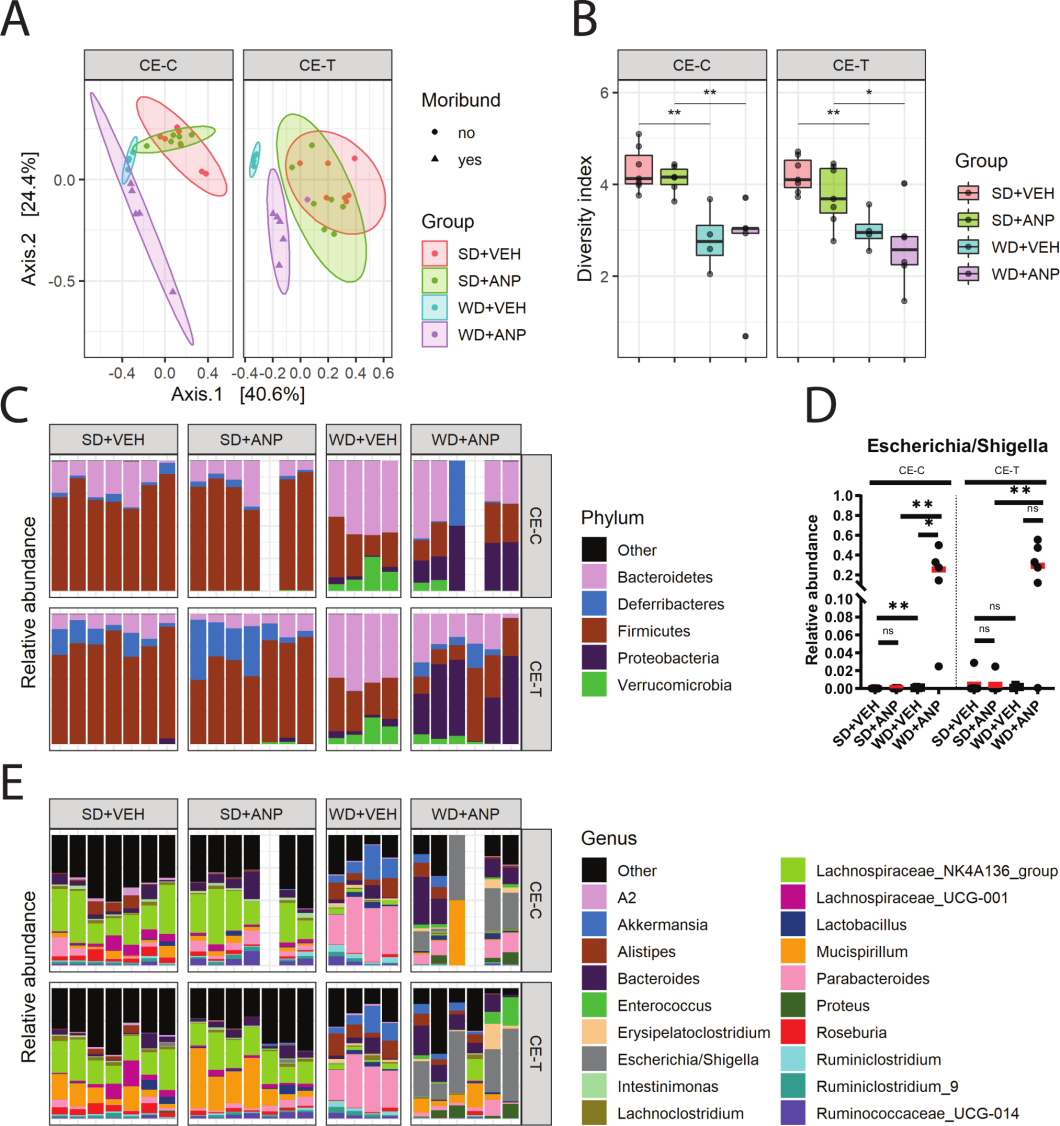
Compositional (16S rRNA gene) analysis of the intestinal microbiota of acute necrotising pancreatitis (ANP) mice. (A) Beta-diversity of cecal content (CE-C) and cecal tissue (CE-T) microbiota measured by weighted UniFrac. A significant difference in microbial composition was observed in Western-type diet (WD)+ANP cecum tissue versus WD+VEH (vehicle) and standard chow diet (SD)+ANP (4–7 mice per group, p=0.003 by Permutation Multivariate Analysis of Variance test). (B) Alpha diversity of CE-C and CE-T microbiota measured by Shannon index. Significant differences were observed in WD+ANP compared with SD+ANP (CE-C: p=0.009; CE-T: p=0.0043 by Mann-Whitney test). No significant differences were observed in WD+ANP versus WD+VEH. (C) Relative bacterial abundance at the phylum level. (D) Relative abundance at the genus level in CE-C and CE-T. (E) Relative abundance of *Escherichia/Shigella* genus in cecum mucosa (p=0.015 by Mann-Whitney test). *p<0.05; **p<0.01; ns, not statistically significant.

Beta-diversity analysis by weighted UniFrac analysis demonstrated significant changes in the composition of cecal luminal and mucosal microbiota in WD+ANP mice as compared with WD mice with saline (vehicle) infusion (WD+VEH) and SD+ANP mice (figure 3A). In contrast, there were no significant differences between SD-fed mice with similar treatments (figure 3A). WD+VEH and WD+ANP mice had comparable alpha diversity measures (measured by the Shannon index) in both lumen and tissue (figure 3B), indicating that the cecal microbiota was disturbed by the WD, independently of pancreatic taurocholate infusion. As demonstrated by the Shannon index, alpha diversity was significantly decreased in WD+ANP compared with SD+ANP mice in both the luminal content and tissue-related microbiota (figure 3B). Relative abundance analysis at the phylum level revealed a profound bloom of Proteobacteria in the luminal content and tissue in WD+ANP mice (p=0.004 in cecal tissue, and p=0.002 in cecal content), but not in SD+ANP treated mice (see figure 3C and online supplementary figure 2). ANP induction with TA infusion as compared with a vehicle infusion control did not affect the microbiota in SD-fed mice.

At the genus level, *Escherichia*/*Shigella* was the most abundant taxa in the cecum content (p=0.016) and tissue (p=0.004) of the WD+ANP group as compared with the SD+ANP group (figure 3D, E). There were no significant differences in *Enterococcus* between the groups.

### The cecum microbiota are functionally disrupted in WD+ANP mice

**Figure 4:**
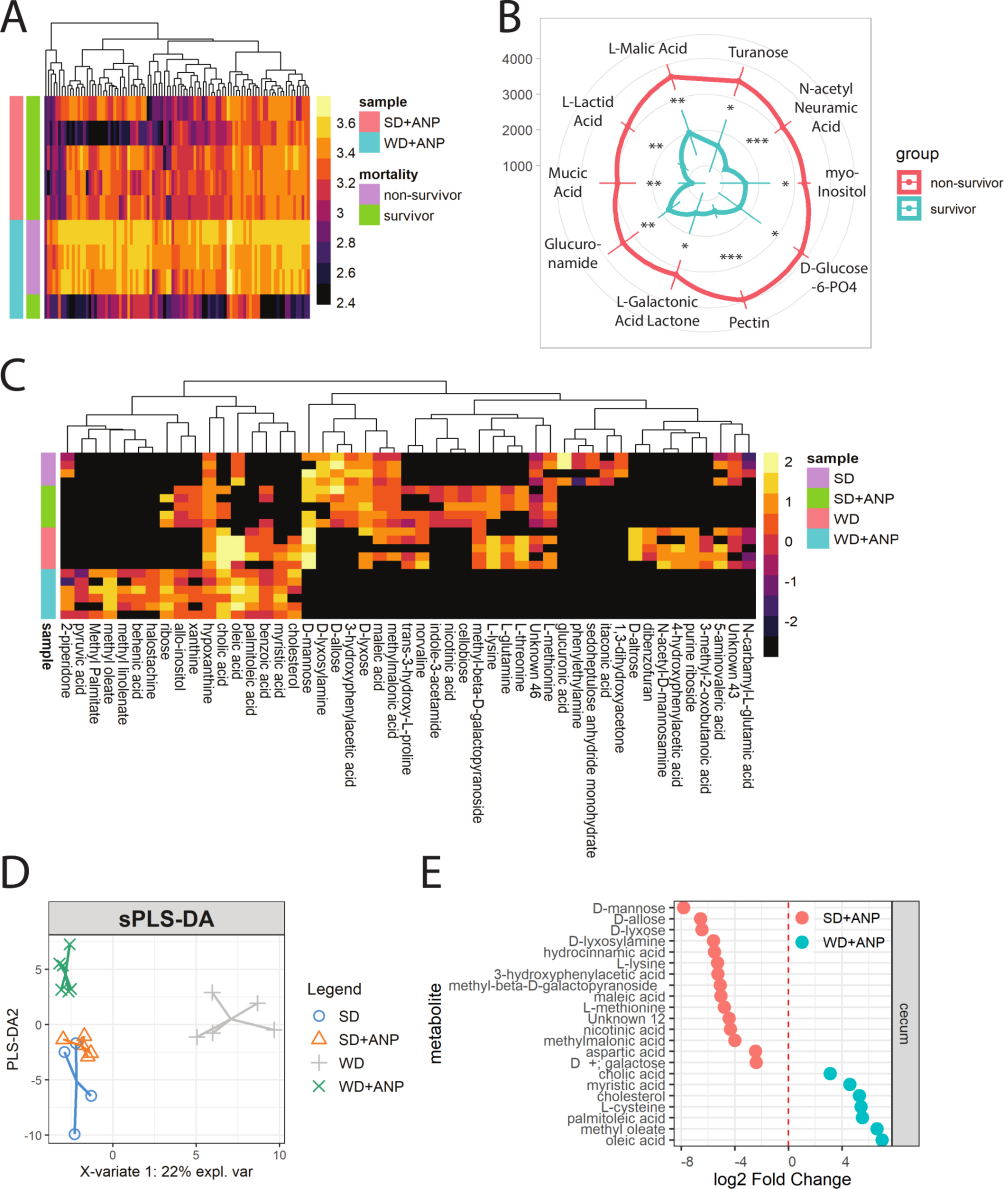
Phenotypic microarray and untargeted metabolomics analysis of intestinal microbiota of acute necrotising pancreatitis (ANP) mice. (A and B) Microbial phenotypic microarray (Biolog) analysis of cecum communities using GENIII plates varied by metabolic substrates. (A) Heatmap of phenotype microarray analysis. There was no statistical difference between standard chow diet (SD)-fed and Western-type diet (WD)-fed ANP mice (p=0.056). When stratified for survival (survivors vs non-survivors), there was a significant increase of metabolic activity in non-survivors (p=0.009 by multiple response permutation procedure). (B) Radial plot of significant carbohydrate substrates showing means with bars representing 95% CI (p<0.05 by Analysis of Variance). (C–E) Untargeted gas chromatography-mass spectrometry (GC-MS) metabolomics analysis of cecum content. (C) Heatmap of differential metabolites between four groups (p<0.05 by Analysis of Variance). (D) Significant test by sparse partial-least squares discriminant analysis (sPLS-DA) showing difference in metabolites of WD pancreatitis mice compared with SD pancreatitis mice (p=0.004) and WD vehicle control mice (p=0.002 by permutations significance test). (E) Univariate analysis shows differential cecal metabolites between SD +ANP and WD +ANP groups (p<0.1 by DESeq analysis). *p<0.05; **p<0.01; ***p<0.001.

**Figure 5:**
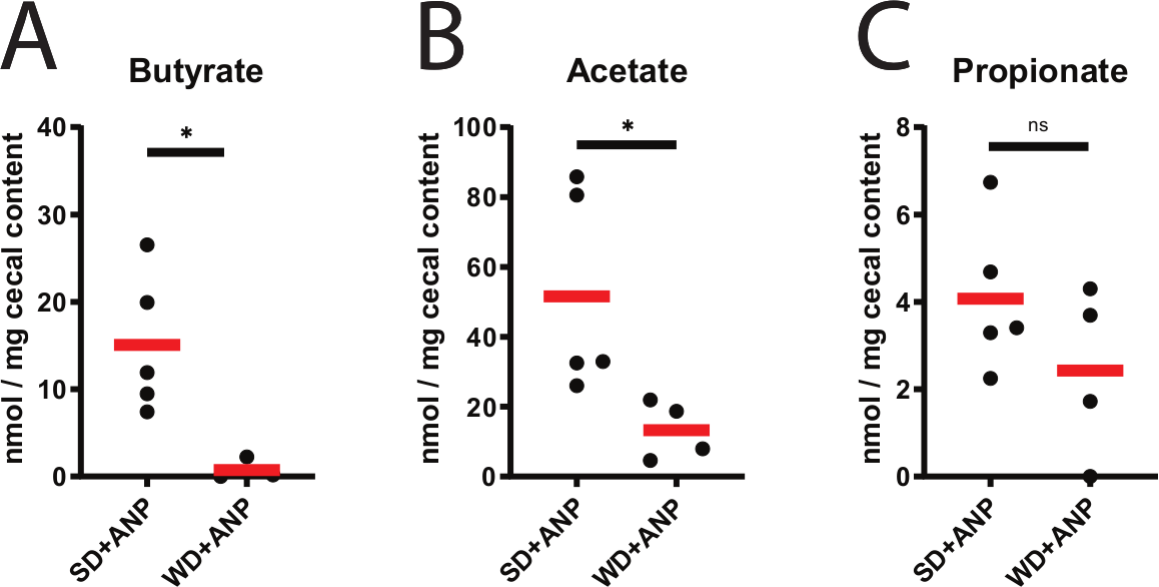
Short-chain fatty acids in the cecum of ANP mice. (A–C) Targeted gas chromatography-mass spectrometry analysis of cecum content for butyrate (p<0.016) (A), acetate (p=0.040) (B) and propionate (p>0.05) (C), all by unpaired t-test. *p<0.05; ns, not statistically significant. ANP, acute necrotising pancreatitis; SD, standard chow diet; WD, Western-type diet.

The ability of the cecal microbial community to use carbon sources and their sensitivity to antimicrobial compounds was investigated using a phenotypic microarray analysis. The results were consistent with the 16S rRNA-based compositional data demonstrating accelerated metabolic respiration under aerobic conditions in gut microbial populations of WD+ANP mice (figure 4A). This was not statistically significant (p=0.056 by multi response permutation procedure), however, when stratified for survival, overall metabolic respiration was significantly increased in non-survivors (p=0.009). Notably, there was significant increased respiration compared with the survivor group in the presence of carbohydrates (turanose, N-acetylneuraminic acid, myoinositol, glucose 6-phospate, pectin), carbohydrate derivatives (L-galactonic acid lactone, glucuronamide, mucic acid, L-lactic acid and L-malic acid) and amino acids (D-serine, glycyl-L-proline, L-arginine, L-aspartic acid, L-histidine and L-serine) (figure 4B and online supplementary figure 3A). Also, non-survivor-derived cecum communities were more resistant to several antimicrobial compounds, including antibiotics such as troleandomycin, rifamycin, lincomycin and vancomycin (see online supplementary figure 3B). Subsequently, the effect of increased metabolic activity of the aerobic cecal microbiota on the luminal metabolic profile was investigated by untargeted metabolomics analysis. ANOVA analysis of all four groups demonstrated 49 metabolites that were differentially distributed among the different groups (figure 4C). Sparse Partial Least Squares Discriminant Analysis (sPLS-DA) demonstrated significant differences of the metabolomes of untreated (SD and WD) and taurocholate-infused (SD+ANP and WD+ANP) groups (figure 4D). Univariate (figure 4E and online supplementary figure 3D, E) analysis demonstrated a cecal depletion of mostly carbohydrates (D-allose, D-lyxose, methyl beta-D-glucopyranoside) and enrichment of long-chain fatty acids (oleic acid, methyl oleate and palmitoleic acid) in WD+ANP compared with SD+ANP groups (figure 4E). Notably, cholic acid was increased in untreated WD-fed mice, as compared with SD-fed mice (see online supplementary figure 3C). Also, ribose, a simple carbohydrate was increased in mice with pancreatitis, independent of the type of diet (see online supplementary figure 3D, E). Additionally, targeted metabolomic analysis for SCFAs demonstrated that cecum acetate and butyrate were significantly depleted (p=0.040 and p=0.016, respectively) in the WD pancreatitis mice compared with SD pancreatitis mice (figure 5A–C). In summary, in this mouse model, WD+ANP mice were at high risk of lethality due to systemic infections that correlated with a bloom of *E. coli* in the cecal lumen and mucosal tissue. This occurred in conjunction with profound changes in the composition and function of the cecal microbiota characterised by a significant decrease in butyrate, amino acids and carbohydrates.

### Antibiotic treatment leads to depletion of *E. coli* in the gut of WD pancreatitis mice and attenuates mortality in this model

**Figure 6:**
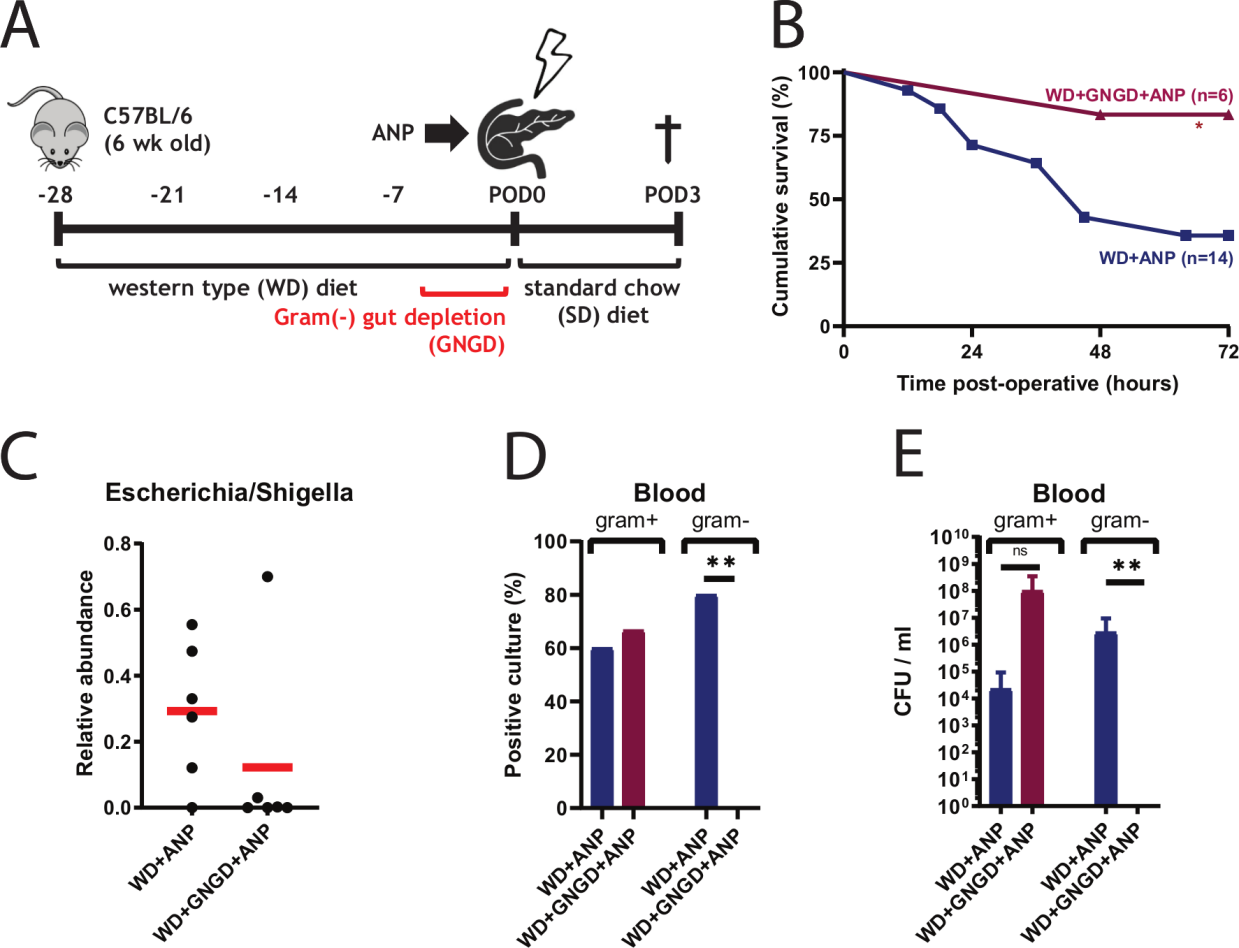
*Escherichia coli* is a major contributor to mortality in Western-type diet (WD)+acute necrotising pancreatitis (ANP) mice. (A) Experimental design for gut depletion of *E. coli*. Gram-negative gut depletion (GNGD) was performed two times per day by oral gavage with non-absorbable antibiotics (neomycin 100 mg/L and polymyxin B 10 mg/L). (B) Kaplan-Meier survival curves demonstrating cumulative survival between treatment groups WD+ANP (n=14) and WD+GNGD+ ANP (n=6). The survival was significantly improved with GNGD (p=0.048, log-rank test). (C) Depletion of *Escherichia/Shigella* in cecum mucosal tissue in five out of six WD+GNGD+ ANP mice, based on 16S rRNA analysis (p=0.132 by Mann-Whitney). (C and D) Culture analysis demonstrates reduction of *E.coli* bacterial load in blood. Significant differences were found for incidence (n=6, 0=0.007 by Fisher exact test) and CFU (p=0.006 by Mann-Whitney test) of Gram-negative cultures, but not for Gram-positive. *p<0.05; **p<0.01; ns, not statistically significant.

To prove that lethal infections associated with ANP in our model are caused by translocating bacteria from the GI tract, mice were treated with oral non-absorbable antibiotics (neomycin and polymyxin B) two times per day for 5 days to deplete the gut of Gram-negative bacteria (GNGD) (figure 6A). Mortality in antibiotic-treated (GNGD) mice decreased from 64% to 17% in WD+ANP mice (p=0.048) (figure 6B), and the relative abundance of *Escherichia/Shigella* in the cecum tissue was strongly reduced in five out of six mice (p=0.132) (figure 6C). None of the blood cultures was positive for Gram-negative bacteria and CFU was significantly decreased (figure 6D, E). The incidence of positive blood cultures was, however, slightly increased as compared with non-antibiotic treated group of WD+ANP, although CFU was not statistically different. In the only moribund mouse in the antibiotic treated group, *Enterococcus casseliflavus* at CFU of 6×10^8^/mL blood was identified.

### Faecal microbiota transplants (FMT) increase mortality and bacterial dissemination in ANP mice

**Figure 7:**
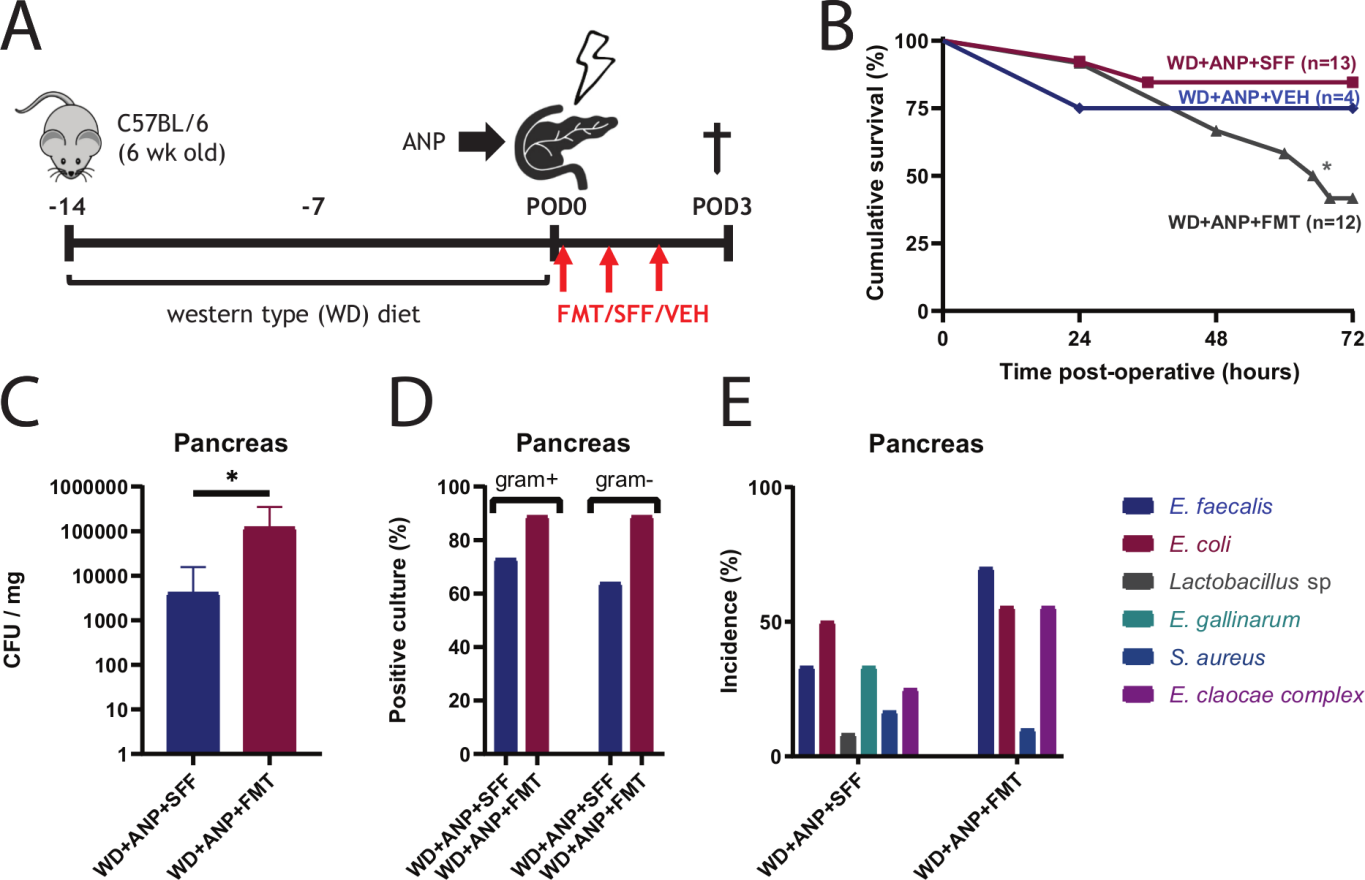
Faecal microbial transplant (FMT) in mice following acute necrotising pancreatitis (ANP) exacerbates bacterial dissemination and worsens survival. (A) Experimental design. Mice fed a Western-type diet (WD) for 2 weeks received either FMT (from healthy mice), sterile faecal filtrate (SFF) or vehicle (VEH) by oral gavage at 0, 24 and 48 hours after ANP induction. (B) Kaplan-Meijer survival curves demonstrating significantly attenuated survival with WD +ANP+ FMT compared with control mice that received SFF (WD +ANP+ SFF) (p=0.047 by log-rank test). (C) Total colony forming units (CFU) were increased in the WD +ANP+ FMT group (p=0.024 by Mann-Whitney test). (D) Incidence of Gram-positive and Gram-negative culture positivity in the pancreas. No significant differences were observed (Fisher exact test). (E) Incidence of cultured bacterial species in pancreatic tissue. *p<0.05.

To investigate the preventative effect of FMT on mortality in this model, a FMT was created from expelled stool from healthy mice. Surprisingly, results from preliminary experiments indicated that FMT accelerated mortality. Therefore, in order to confirm this, a less lethal model of ANP was established by reducing the days of WD feeding to 14 days, before ANP induction. Subsequently, at 1, 24 and 48 hours following the surgical procedure, mice were administered a FMT or sterile faecal filtrate (SFF) through oral gavage and were monitored for survival during 72 hours postoperatively (figure 7A). Mortality was significantly increased in pancreatitis mice that received FMT compared with mice that received SFF (figure 7B). Bacterial translocation was increased with FMT, as demonstrated by an increase of total CFU of pancreatic tissue cultures and increased incidence of both Gram-negative and Gram-positive pathogenic bacteria (figure 7C, D). In blood, although no statistical difference in CFU was observed, the incidence of Gram-positive cultures was increased (see online supplementary figure 4A, B). In contrast to previous experiments, a shift from *E. coli* being the predominant pathogen in the SFF group, to *E. faecalis* in the FMT group, was noticed in both pancreatic tissues as blood (figure 7E and online supplementary figure 4C). Furthermore, 16S rRNA gene amplicon analysis of the administered FMT shows that the genera of the three dominating isolated pathogens (*E. coli*, *E. cloacae complex* and *E. faecalis*) only account for approximately 0.02% of total bacteria (online supplementary figure 4D) that are present in the FMT.

### Oral and intraperitoneal butyrate reduces mortality and *E. coli* dissemination in ANP mice

**Figure 8:**
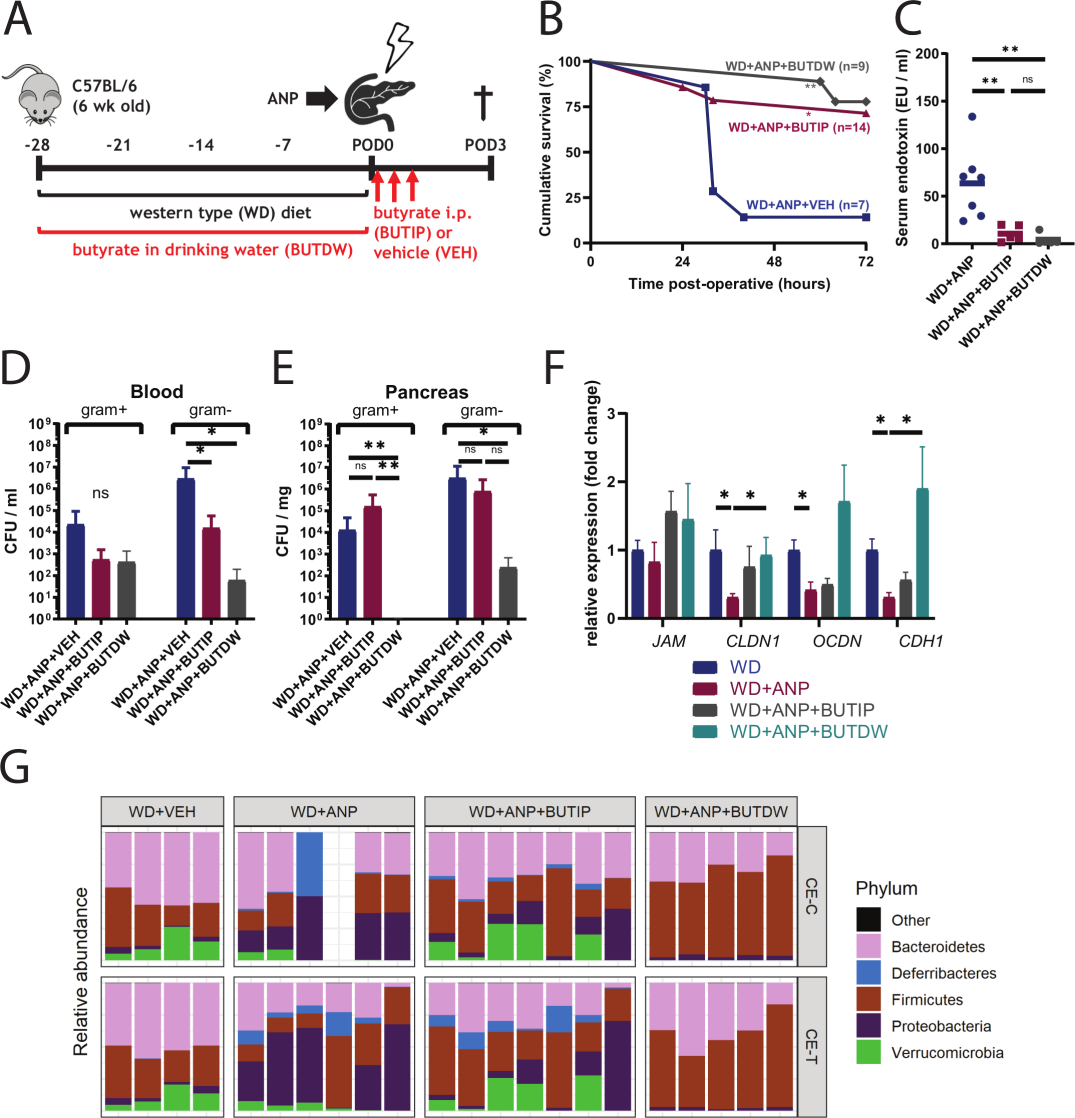
Oral and systemic butyrate supplementation reduces mortality and bacterial dissemination in Western-type diet (WD)+acute necrotising pancreatitis (ANP) mice. (A) Butyrate was supplemented either preoperatively in the drinking water (BUTDW, 100 mM) for 4 weeks or systemically with intraperitoneal injections (BUTIP, 40 mM, 500 µL) at 0, 7 and 14 hours postoperatively. (B) Kaplan-Meijer survival curves demonstrating significantly reduced mortality with both oral (WD+ANP+BUTDW, n=9, p=0.002) and systemic (WD+ANP+BUTIP, n=14, p=0.019) butyrate supplementation. Survival curves are analysed using log-rank test. (C) Serum endotoxin levels are significantly attenuated by BUTDW (n=5, p=0.003 by Mann-Whitney) and BUTIP (n=5, p=0.012 by unpaired t-test) (D, E) Culture analysis of blood (D) and pancreas (E). p=0.016 of Gram-negative bacteria in BUTIP versus vehicle (VEH) and p=0.028 in BUTDW versus VEH in blood (D). p=0.012 of Gram-negative bacteria in BUTIP versus VEH, and p=0.088 in BUTIP versus VEH in pancreas (E). (F) Expression of tight junction genes indicates increased expression of *CLDN1* (n=5, p=0.016) and *CHD1* (p=0.032) in the cecum tissue of BUTDW mice versus the untreated control group (WD+ANP). (G) 16S rRNA analysis of relative abundance. Relative abundance at the phylum level demonstrating depletion of Proteobacteria in the BUTDW group to the level of WD+VEH (p=0.008 vs WD+ANP in cecum lumen). Depletion of Proteobacteria was also observed in WD+ANP+BUTIP mice but did not reach statistical significance. *p<0.05; **p<0.01; ns, not statistically significant.

Given the depletion of butyrate in the gut of WD+ANP group of mice, butyrate was supplemented into the drinking water or alternatively administered to mice via intraperitoneal injection. Butyrate, a gut microbiota-derived metabolite, is a critical carbon source for colonic enterocytes^20 21^ suggesting that it may be protective against gut barrier impairment. It has been demonstrated that butyrate enhances the intestinal barrier by regulating the assembly of tight junctions.^22^ It is also involved in the regulation of gut microbiota pathogenesis^23^ providing a protective role against invading pathogens.^24^ Therefore, butyrate supplementation was tested for its ability to rescue mice from lethal ANP. Supplemented butyrate was added to either the drinking water (100 mM) of WD mice or administered by intraperitoneal injection (500 µL, 40 mM) as designed on figure 8A. Butyrate provided in the drinking water (BUTDW) increased the gut butyrate concentration (see online supplementary figure 5A). Both oral and systemic treatment significantly increased survival in WD pancreatitis mice (figure 8B) with a greater protective effect demonstrated with oral butyrate (n=9, p=0.002 for oral and n=14, p=0.019 for systemic butyrate, log-rank test). Reduction of bacterial translocation and gut permeability was demonstrated as judged by a decrease in serum endotoxin (figure 8C) levels and Gram-negative bacterial dissemination in blood (figure 8D) with both butyrate treatments, however, only orally supplemented butyrate prevented pancreatic infection with *E. coli* (figure 8E). The expression of genes involved in paracellular junctions was attenuated in ANP mice (*CLDN1*, p=0.016; *OCDN*, p=0.032; *CHD1*, p=0.016) which, in contrast, were significantly increased with oral butyrate treatment *CLDN1* (p=0.016) and *CHD1* (p=0.032) with a trend towards an increase with IP butyrate treatment (figure 8F). 16S rRNA analysis demonstrated that oral butyrate shifted the composition of cecal microbiota towards WD+VEH control profile (see online supplementary figure 5B) and completely prevented cecal lumen and tissue colonisation with Proteobacteria (figure 8G and online supplementary figure 5C) and specifically, *Escherichia*/*Shigella* (see online supplementary figure 5D).

Finally, mice were injected with TSA, a potent class I and II HDAC inhibitor with a half-life time comparable to sodium butyrate, following ANP induction. TSA treatment did not reduce mortality, indicating that the systemic butyrate effect on survival was independent of HDAC inhibition (see online supplementary figure 6).

### The phylum Proteobacteria and the genera *Escherichia/Shigella* and *Streptococcu*s are increased in patients with pancreatitis in association with a decrease in butyrate-producing strains

**Figure 9:**
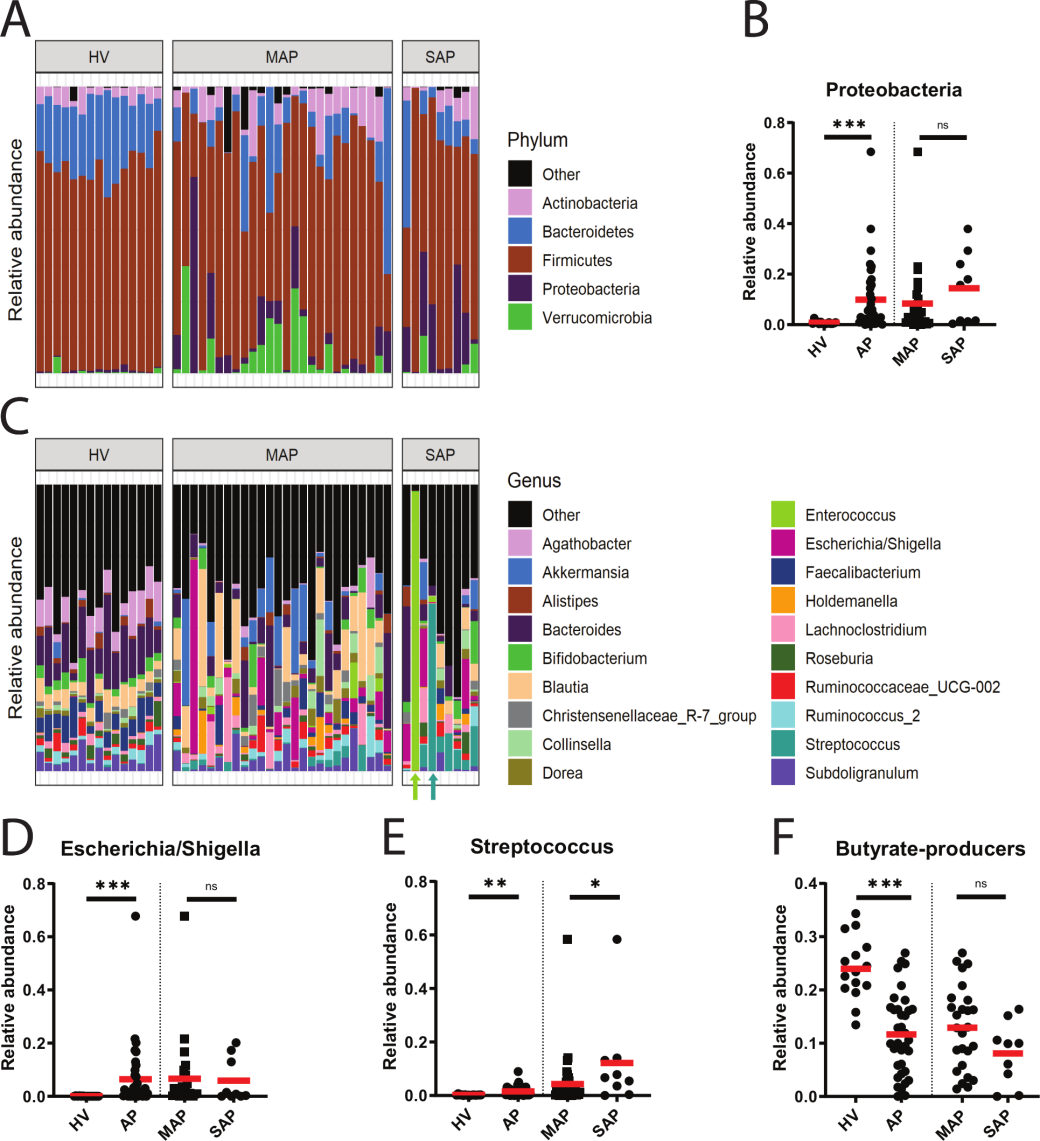
16S rRNA analysis of human samples. (A) Relative abundance of microbiota at the phylum level in faeces of 15 healthy volunteers (HV) and 36 patients with acute pancreatitis (AP) including 26 patients with mild AP (MAP) and nine patients with severe AP (SAP). (B) Proteobacteria was significantly increased in AP compared with HV (p=0.0002). No differences were observed between MAP and SAP (p=0.224, both by Mann-Whitney test). (C) Relative abundance of microbiota at the genus level. *Blautia*, *Akkermansia* and *Escherichia/Shigella* were among the dominating species in MAP while *Escherichia/Shigella, Enterococcus and Streptococcus*—in patients with SAP. In two patients with SAP, a near monoculture community was observed at the genus level (*Enterococcus* and *Streptococcus*) as indicated by arrows. (D) Relative abundance of *Escherichia/Shigella* was significantly increased in AP compared with HV (p<0.001). No differences between MAP and SAP (p=0.540, both by Mann-Whitney test). (E) Relative abundance of *Streptococcus* was significantly increased in AP compared with HV (p=0.003), and in SAP compared with MAP (p=0.047, both by Mann-Whitney test). (F) Relative abundance of butyrate producers was significantly decreased in patients with AP as compared with HV (p<0.001). When stratified for severity, no statistical difference was found between MAP and SAP (p=0.100, both by unpaired t-test). *p<0.05; **p<0.01; ***p<0.001, ns, not statistically significant.

Next, faecal samples from patients with pancreatitis were analysed and compared with healthy volunteers (HV). Fifteen HV and 35 patients with AP including 26 patients with MAP and 9 patients with SAP were included in the study. 16S rRNA analysis demonstrated that the microbiota composition of AP and HV groups differed (p=0.001), with a decrease of alpha diversity (p<0.0001) in AP (see online supplementary figure 7). The phylum Proteobacteria was present at high frequency in patients with pancreatitis (figure 9A, B) with a 13-fold increase as compared with HV. At the genus level, there were 100-fold and 10-fold increase in *Escherichia/Shigella* (figure 9C, D) and *Streptococcus* (figure 9E), respectively. Most importantly, butyrate producers were significantly decreased in patients with AP (both MAP and SAP) as compared with control HV (twofold) (figure 9F).

## Discussion

Among patients with AP, secondary infection of the gland with necrosis is a potentially fatal clinical complication that typically necessitates radiological, endoscopic or surgical intervention.^25–27^ Despite recent advancements in the treatment of infected necrotising pancreatitis—such as the step-up approach^28^ and minimal invasive endoscopic procedures^29^—it is still a life-threatening condition with high morbidity and mortality, requiring long hospital—and ICU admissions.^30^ To date, there are no effective prophylactic strategies to prevent infectious complications in AP. The present study demonstrated that diet-induced disturbances in the gut microbiota aggravated infections in experimental necrotising pancreatitis, evidenced by increased bacterial dissemination, systemic inflammation and mortality in mice that were fed a WD, as compared with control mice on a SD. These findings were associated with a decrease in bacterial diversity and the butyrate-producing capacity of the gut microbiota. Surprisingly, treatment with faecal transplants (FMT) increased mortality and bacterial translocation, perhaps owing to the known altered intestinal permeability in patients^31^ and animal models of AP.^32–36^ It is possible that gavaging FMT in this model could have resulted in some of its members directly entering the pancreatic duct. Prior work by others has demonstrated that oral gavage of bacteria can cause pancreatic contamination via reflux into the pancreatic duct.^37^ While this is certainly a possibility in the present study, given the low relative abundance of the contaminating pancreatic pathogens present in the introduced FMT, compared with the extremely high dose used in the study by Pushalkar, this mechanism seems less likely. An alternative explanation for the failure of FMT to protect in this model may be a function of the known perturbations in the intestinal microenvironment created by a WD and hence could have enhanced the virulence of the administered pathogens present in the FMT. Further experiments are needed to clarify the mechanism by which FMT worsened outcome in this model.

In contrast, supplementation of butyrate—both oral prophylactic treatment and systemically postpancreatitis treatment—showed a protective effect with a clear reduction of bacterial dissemination, serum endotoxin levels and mortality, and an increase of expression of genes involved in paracellular (tight) junctions. Butyrate promotes the enhancement of the intestinal barrier^38^ thus preventing bacterial dissemination and endotoxin permeation. Additionally, butyrate may enhance immune clearance mechanisms at the systemic level via Interferon Regulatory Factor 3 (IRF3) as has been recently demonstrated.^39^

We have previously shown that in both humans and mouse models of acute physiological stress, the gut microbiome becomes dramatically altered in composition and function resulting in a major loss of the anaerobic populations (>90%), depletion of SCFAs (>90%) and overgrowth of Proteobacteria (ie, *E. coli*).^5 39–41^ Our current data suggest that diet-independent enrichment of ribose, a substrate that can serve as sole energy source for *E. coli*,^42^ may in part explain gastrointestinal overgrowth of *E. coli* in our model. The precise mechanisms for this response, however, remain unknown and under investigation.

This study is in agreement with a growing body of evidence demonstrating the protective role of the healthy intestinal microbial ecosystem to defend against both low abundance endogenous microbiota that can bloom under conditions such as ANP as well as against invading exogenous pathogens.^5 43–45^ Butyrate, a SCFA produced by gut commensals, is the main energy source for colonocytes, in contrast to long-chain fatty acids that are only poorly used.^46^ Our study showed a highly significant depletion of SCFAs, carbohydrates and amino acids, which are associated with WD feeding and ANP. Conversely, there was an increase of long-chain fatty acids oleic acid, methyl oleate and palmitoleic acid. Based on these results, one might hypothesise that a lack of utilisable metabolic substrates and an increase of bile acids lead to atrophy, inflammation and an increased intestinal permeability. Providing a critical energy source likely contributes to the attenuated bacterial dissemination and mortality that was observed with butyrate supplementation. This is supported by recent evidence that shows that oral butyrate supplementation partially reversed increased gut permeability and dysbiosis caused by feeding a high-fat WD.^47 48^

Recent studies have shown that SCFAs can provide protection against *Clostridium difficile* collitis^49^ and colonisation with *Salmonella* 50 and *Candida albicans*.^51^ Butyrate regulates macrophage function through HDAC inhibition and drives them towards an antimicrobial phenotype during maturation.^24 52^ These immunological antimicrobial effects, mediated by HDAC inhibition, might underlie the mechanism for attenuation of ANP with systemic butyrate in our model. However, treatment with TSA, a potent HDAC inhibitor, did not provide protection in our model. Selectivity of butyrate and TSA for HDAC classed differ, demonstrated by the fact that intestinal epithelial cells respond differently in their presence. Another explanation is the added effects of butyrate on the immune response, gut permeability and locally on the gut microbiota. Further work is needed, however, to fully rule out the beneficial effect of HDAC inhibitors in this model.

In the present study, an attempt was made to define the role of the microbiota and their metabolites in the pathogenesis of ANP and secondary pancreatic infection. Furthermore, adding multiple antibiotics to these patients over the long course of illness can add to the already comprised state that leads to a bloom in multidrug-resistant organisms that complicate the overall course of the disease. In 1995, Luiten *et al* 53 published a clinical trial where they randomised 102 patients with SAP to either standard treatment or standard treatment with selective oral decontaminating agents for Gram-negative bacteria (norfloxacin, colistin, amphotericin, cefotaxime) and showed reduced mortality and infections in the treatment group. Although it seems effective, the mechanism of action of selective digestive decontamination in AP remains poorly described. However, the increase of Gram-positive blood-stream infections in this study demonstrates the possibility of a classic trade-off scenario with the overuse of antibiotics. Selective pressure for low-abundance Gram-positive pathogens can cause overgrowth of these pathogens, induce multidrug-resistance and unintentionally cause lethal infections. This indicates that there is a clear need for microbiota modulating therapies that are supporting the native, commensal microbial communities, as opposed to antibiotics that induce multidrug-resistant pathobionts.^5^

Recently, the US Food and Drug Administration issued a safety alert for the use of FMT after reports of serious adverse effects, including one death, due to infections with multidrug-resistant bacteria.^54^ Our findings further indicate that extreme caution is warranted when considering administration of live bacteria in a clinical or experimental setting to this severely ill patient population. The therapeutic use of so-called postbiotics—bacterial metabolites or components that are released when bacteria are lysed—are becoming increasingly popular targets because of their potential potency and beneficial safety profile.^55 56^

In summary, results from the present study provide compelling evidence that diet and its interaction with the intestinal microbiota can have a profound effect on the course and outcome of ANP. Results from this study also define the limits and risks of using FMT and antibiotics to prevent mortality from ANP in WD mice. Novel approaches to prevent the progression of patients who present with clinical pancreatitis to infected pancreatic necrosis can be informed by this study, which suggests that nutrient availability—amongst other postbiotics such as butyrate—to the microbiota may be a more rational strategy.

## Data Availability

Data are available upon reasonable request.
