# Supplementary material for: Western-type diet influences mortality from necrotising pancreatitis and demonstrates a central role for butyrate
Source: Gut. 2020 Sep 1;70(5):915–27. doi: 10.1136/gutjnl-2019-320430 (PMC7917160; doi:10.1136/gutjnl-2019-320430)
Supplement: Supplementary data [file gutjnl-2019-320430supp001.pdf]

Online Supplementary Methods

Western-type diet influences mortality from necrotizing pancreatitis and demonstrates a central role for butyrate

Fons F van den Berg [1,3], Demi van Dalen [2,3], Sanjiv K Hyoju [3], Hjalmar C van Santvoort [4,5], Marc G Besselink [1], W Joost Wiersinga [6,7], Olga Zaborina [3], Marja A Boermeester [1], John C Alverdy [3]

Surgical procedures

Before surgery, mice were anesthetized with an intraperitoneal injection of ketamine, 100 mg/kg body weight (BW) and xylazine (5 mg/kg BW) (Henry Schein Animal Health, Dublin, OH). A pre-emptive injection of bupinorphrine (0.1 mg/ kg BW) and meloxicam (1 mg/kg BW) was given. The posterior side of the duodenum was exposed by a 1 cm midline laparotomy just below the xipoid process. The papilla of Vater was identified and a puncture was created with a 26G needle at the opposite side of the duodenum. A 30G blunt needle connected to a syringe pump (Harvard apparatus, Holliston, MA) with PE-10 tubing (Fisher Scientific, Waltham, MA) was inserted into the common bile duct. The cannula was temporarily fixated with an 8-0 prolene suture, and a micro-clamp was placed at the proximal hepatic duct. The pancreas was retrograde infused for 10 min with either 50 ul saline (control mice) or 4% sodium taurocholate hydrate (pancreatitis mice) with 1% methyl-blue (Sigma-Aldrich, Saint-Louis, MO). The cannula, micro-clamp and ligature were removed and the puncture hole closed with a single 8-0 prolene suture. The abdominal wall and skin were closed with, respectively with 6-0 vicryl and 5-0 nylon interrupted sutures. Post-operatively, injections of bupinorphrine were given every 12 hours for at least 48 hours. All procedures were performed under sterile conditions according to local guidelines and policies.

Bacterial dissemination

Blood and organ tissues (pancreas, liver, spleen, mesenteric lymph nodes and lung) were weighted, homogenized in saline and 10-fold serial dilutions were plated on selective media for gram-positive (Colombia CNA with 5% sheep blood) and Gram-negative bacteria (Difco™ MacConkey Agar, Becton Dickinson, Franklin Lakes, NJ). Colony forming units were counted after 24 hours of incubation at 37° degrees Celsius and normalized by volume (blood) or weight (tissue). Positive blood and pancreas tissues culture were sent to the Clinical Microbiology laboratory for identification using a Vitek MS (bioMérieux, Inc., Durham, NC). Isolates were cultured in liquid TSB medium and stored in 10% glycerol at -80 degrees Celsius.

Histology

Pancreatic tissues at the pancreatic head were fixated in formalin for 24 hours following sacrifice. The slides were stained with an H&E staining and scored for parenchymal necrosis and inflammation according to criteria of Schmidt by an experienced pathologist.[1]

Serum laboratory markers

Amylase was detected in serum using the UCSC Clinical Laboratory via routine methods. Serum interleukin-6 (IL-6), interleukin-1b (IL-1b) and Tumor necrosis factor alpha (TNFα) were measured using commercial enzyme immune assay (mouse IL-6, OptEIA, Becton Dickinson, Franklin Lakes, NJ; Mouse IL-6 Ready-SET-Go! and Mouse TNF alpha Ready-SET-Go! kits, Invitrogen, Carlsbad, CA) according to instructions. Endotoxin was measured using a chromogenic endotoxin detection assay (Pierce Chromogenic Endotoxin Quant Kit, Thermo Scientific, Waltham, MA).

Bacterial community Phenotype Microarray analysis

The metabolic activity of the intestinal bacterial community was determined as previously described.[2] Briefly, cecal contents were homogenized in saline, centrifuged at 100G for 3 min and the supernatant diluted to an OD600 of 0.25. 50 ul of the sample was added to the IF-A inoculating fluid (Biolog, Haward, CA). GEN-III plates (Biolog, Haward, CA) were incubated with 100 ul inoculating fluid and incubated at an Omnilog incubator/reader for 22 hours at 37 degrees Celsius. Data were analyzed with R statistical software and the “opm” package.[3] The area under the curve (ACU) of the growth curve wag aggregated from the raw data using the function ‘do_aggr’. Pairwise comparisons of the substrates by ANOVA between the experimental groups were done with the function “opm_mcp”. Group comparisons by Multiple Response Permutation Procedure (MRPP) with 999 permutations were done with the function “mrpp” of the “vegan” package.[4]

Targeted GC-MS analysis of SCFAs

Short chain fatty acids were assayed in approximately 50 mg of mouse cecal contents by first homogenizing the sample in 600 ul nuclease free water followed by centrifugation at 15,000G for 10 min. 100 ul of 50% sulfuric acid (Sigma-Aldrich, Saint-Louis, MO) was added to the supernatant of each sample and incubated overnight at RT. 5 ul of 0.88% Isobutyric acid was added as internal control. SCFAs were extracted using 500 ul diethyl ether (Fisher Scientific, Waltham, MA) and repeated two more times. The samples were silylated overnight with N-tert-Butyldimethylsilyl-N-methyltrifluoroacetamide with 1% tert-Butyldimethylchlorosilane and the derivates were processed for acetate, propionate and butyrate levels on an 5977A Single Quad and 7890B GC (Agilent, Santa Clara, CA). The measurements were normalized by weight.

Untargeted GC-MS metabolomics analysis

100 ul of ice-cold HPLC grade water was added, homogenized and centrifuged at 16.000 g for 20 min at 4°. Supernatant was collected and 450 ul ice cold HPLC- grade methanol was added, homogenized and centrifuged. The supernatants were combined and filtered through a 0.22 um filter. 5 ul of 3 mg/ml myristic acid d27 (Sigma Aldrich, Saint-Louis, MO) was added as internal control. The samples were evaporated in a Vacufuge (Eppendorf, Hamburg) and the residue stored at -80°C until further use.

Samples were derived by adding 50 ul of 20 mg/ml methoxyamine (Sigma) in pyridine. Samples were incubated at 37°C for 90 minutes in a shaking incubator. Next, 100 ul of MSTFA + 1% TMC (Sigma) was added and incubated at 37°C for 30 min. before analysis.

The samples were analyzed using a high-resolution accurate mass 7200B QTOF GC-MS Agilent, Santa Clara, CA) with a mass range 35-800, acquisition rate 6 spectra/s. Samples were randomized in batches of 5, and injected at a 25:1 split ratio. A temperature program of 70°C for 5 min was used, ramped at 5°C/min until 320°C, and held for 3 min. A blanc sample containing the solvent (pyridine) was run between each sample to clean the column of any residue. The chromatograms were processed (peakpicking, definition of pseudospectra and annotation) using R (R Core Team, 2014) with the “runGC” function of the metaMS package.[5] The resulting pseudospectra were annotated using the commercial Fiehn GC/MS Metabolomics RTL Library[6]. Features with matching factors ≥60% were positively identified.

16S rRNA gene sequencing of human fecal samples

DNA was extracted from fecal material using a repeated bead beating protocol[7] (method 5). DNA was purified using Maxwell RSC Whole Blood DNA Kit. 16S rRNA gene amplicons were generated using a single step PCR protocol targeting the V3-V4 region.[8] PCR products were purified using Ampure XP beads

and purified products were equimolar pooled. The libraries were sequenced using a MiSeq platform using V3 chemistry with 2x251 cycles.

Forward and reverse reads were truncated to 240 and 210 bases respectively and merged using USEARCH.[9] Merged reads that did not pass the Illumina chastity filter, had an expected error rate higher than 2, or were shorter than 380 bases were filtered. Amplified Sequence Variants (ASVs) were inferred for each sample individually with a minimum abundance of 4 reads.[10] Unfiltered reads were than mapped against the collective ASV set to determine the abundances. Taxonomy was assigned using the RDP classifier[11] and SILVA[12] 16S ribosomal database V132.

RNA isolation and RT-qPCR

Cecum tissues used for RNA isolation were collected in RNAlater and stored at -80 degrees Celsius until analysis. 20-30 mg of tissue was homogenized using a Precellys 24 tissue homogenizer (Bertin instruments, Montigny-le-Bretonneux, France). Total RNA was isolated using the NucleoSpin kit (Macherey-Nagel, Dueren, Germany) according to the manufacture's manual. Genomic DNA was removed using the Heat&Run gDNA removal kit (ArcticZymes AS, Tromsø, Norway) following the instructions. 500 ng of RNA was reverse transcripted using the SuperScript III Reverse Transcriptase (Thermo Fisher Scientific Inc., Waltham, MA, USA). Quantitative PCR was done using Sensifast SYBR No-ROX Mix (Bioline, London, UK) on a Lightcycler 380 Real-Time PCR System (Roche Applied Science, Penzberg, Germany). The following primers were used:

**Gene**	**Forward sequence (5'-3')**	**Reverse sequence (5'-3')**
B2M	CCC CAC TGA GAC TGA TAC ATA CG	CGA TCC CAG TAG ACG GTC TTG
CHD1	CCT GTC TTC AAC CCA AGC AC	CAA CAA CGA ACT GCT GGT CA
OCLN	CTC TCA GCC AGC GTA CTC TT	CTC CAT AGC CAC CTC CGT AG

CLDN1	CACTCCCAGACTCCACCACC	CGATCCATCCCAGAGAAGCC
JAM	ACT GCT CAA TCT GAC GTC CA	ATA GGG AGC TGT GAT CTG GC

Ct values were calculated using LinRegPCR software.[13] Relative gene expression was performed using the delta-delta Ct method with b2-microglobulin (B2M) as reference.[14]

References

1. Schmidt J, Rattner DW, Lewandrowski K, et al. A better model of acute pancreatitis for evaluating therapy. *Ann Surg* 1992;215(1):44-56.
2. Hyoju SK, Zaborin A, Keskey R, et al. Mice Fed an Obesogenic Western Diet, Administered Antibiotics, and Subjected to a Sterile Surgical Procedure Develop Lethal Septicemia with Multidrug-Resistant Pathobionts. *mBio* 2019;10(4) doi: 10.1128/mBio.00903-19
3. Vaas LA, Sikorski J, Hofner B, et al. opm: an R package for analysing OmniLog(R) phenotype microarray data. *Bioinformatics* 2013;29(14):1823-4. doi: 10.1093/bioinformatics/btt291
4. Oksanen J, Blanchet FG, Friendly M, et al. vegan: Community Ecology Package. R package version 2.5-6. 2019
5. Wehrens R, Weingart G, Mattivi F. metaMS: an open-source pipeline for GC-MS-based untargeted metabolomics. *J Chromatogr B Analyt Technol Biomed Life Sci* 2014;966:109-16. doi: 10.1016/j.jchromb.2014.02.051
6. Kind T, Wohlgemuth G, Lee DY, et al. FiehnLib: mass spectral and retention index libraries for metabolomics based on quadrupole and time-of-flight gas chromatography/mass spectrometry. *Anal Chem* 2009;81(24):10038-48. doi: 10.1021/ac9019522
7. Costea PI, Zeller G, Sunagawa S, et al. Towards standards for human fecal sample processing in metagenomic studies. *Nat Biotechnol* 2017;35(11):1069-76. doi: 10.1038/nbt.3960
8. Kozich JJ, Westcott SL, Baxter NT, et al. Development of a dual-index sequencing strategy and curation pipeline for analyzing amplicon sequence data on the MiSeq Illumina sequencing platform. *Appl Environ Microbiol* 2013;79(17):5112-20. doi: 10.1128/AEM.01043-13
9. Edgar RC. Search and clustering orders of magnitude faster than BLAST. *Bioinformatics* 2010;26(19):2460-1. doi: 10.1093/bioinformatics/btq461 [published Online First: 2010/08/17]
10. Edgar R. UNOISE2: improved error-correction for Illumina 16S and ITS amplicon sequencing: bioRxiv, 2016.
11. Wang Q, Garrity GM, Tiedje JM, et al. Naive Bayesian classifier for rapid assignment of rRNA sequences into the new bacterial taxonomy. *Appl Environ Microbiol* 2007;73(16):5261-7. doi: 10.1128/aem.00062-07 [published Online First: 2007/06/26]
12. Quast C, Pruesse E, Yilmaz P, et al. The SILVA ribosomal RNA gene database project: improved data processing and web-based tools. *Nucleic Acids Res* 2013;41(Database issue):D590-6. doi: 10.1093/nar/gks1219
13. Ramakers C, Ruijter JM, Deprez RH, et al. Assumption-free analysis of quantitative real-time polymerase chain reaction (PCR) data. *Neurosci Lett* 2003;339(1):62-6. doi: 10.1016/s0304-3940(02)01423-4
14. Livak KJ, Schmittgen TD. Analysis of relative gene expression data using real-time quantitative PCR and the 2(-Delta Delta C(T)) Method. *Methods* 2001;25(4):402-8. doi: 10.1006/meth.2001.1262
