# Supplementary material for: Western-type diet influences mortality from necrotising pancreatitis and demonstrates a central role for butyrate
Source: Gut. 2020 Sep 1;70(5):915–27. doi: 10.1136/gutjnl-2019-320430 (PMC7917160; doi:10.1136/gutjnl-2019-320430)
Supplement: Supplementary data [file gutjnl-2019-320430supp002.pdf]

**Supplementary Table 1** Panel of butyrate-producing taxa at the genus level
Alistipes
Anaerostipes
Butyricicoccus
Butyricimonas
Butyrivibrio-010
Coprococcus_1
Coprococcus_2
Coprococcus_3
Eubacterium
Faecalibacterium
Flavonifractor
Odoribacter
Oscillibacter
Pseudoflavonifractor
Roseburia
Ruminococcus_2
Subdoligranulum
