# Supplementary material for: Western-type diet influences mortality from necrotising pancreatitis and demonstrates a central role for butyrate
Source: Gut. 2020 Sep 1;70(5):915–27. doi: 10.1136/gutjnl-2019-320430 (PMC7917160; doi:10.1136/gutjnl-2019-320430)
Supplement: Supplementary data [file gutjnl-2019-320430supp003.pdf]

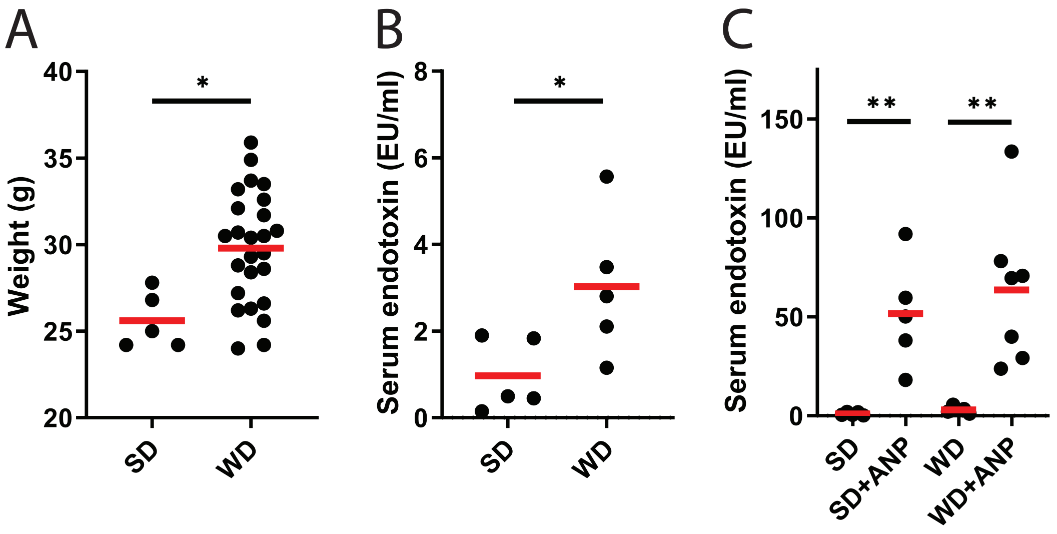

Supplementary Figure 1 Effect of western type (WD) diet on body weight (p=0.011) (A) and intestinal permeability measured by serum endotoxin (B), compared to a standard chow (SD) diet (p=0.032, both by Mann-Whitney test). (C) Serum endotoxin levels of SD and WD are increased at 24 hours after pancreatitis induction, compared to untreated SD and WD mice (respectively, p=0.003 and p=0.05 by unpaired t-test). *p<0.05; **p<0.01.

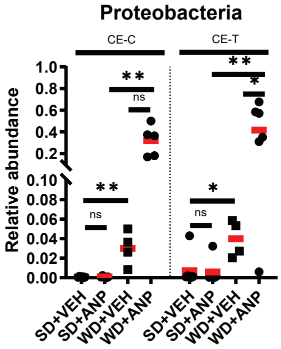

Supplementary Figure 2 Relative abundance of Proteobacteria in cecum tissue (CE-T) (p=0.004) and content (CE-C) (p=0.002, both by Mann-Whitney test). *p<0.05; **p<0.01, ns, not statistically significant.

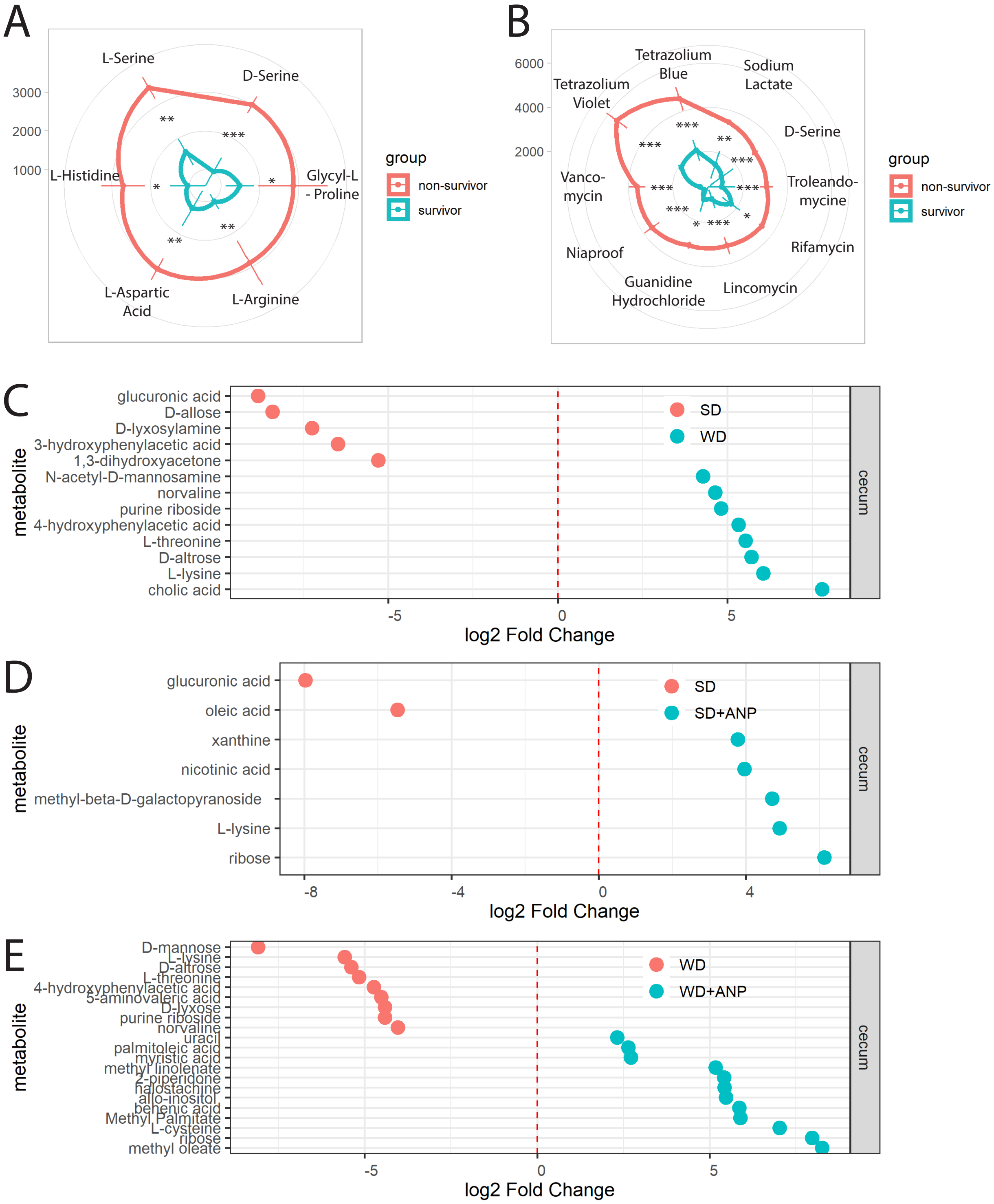

Supplementary Figure 3 (**A-B**) Radial plot of significant phenotype microarrays using Biolog GENIII plates showing means with bars representing 95% CI (p<0.05 by ANOVA) of aminoacids (**A**) and antimicrobials (**B**) (**D-E**) Univariate metabolomics analyses showing differential abundant metabolites (p<0.1 by DESeq analysis) between experimental groups. *p<0.05; **p<0.01; ***p<0.001.

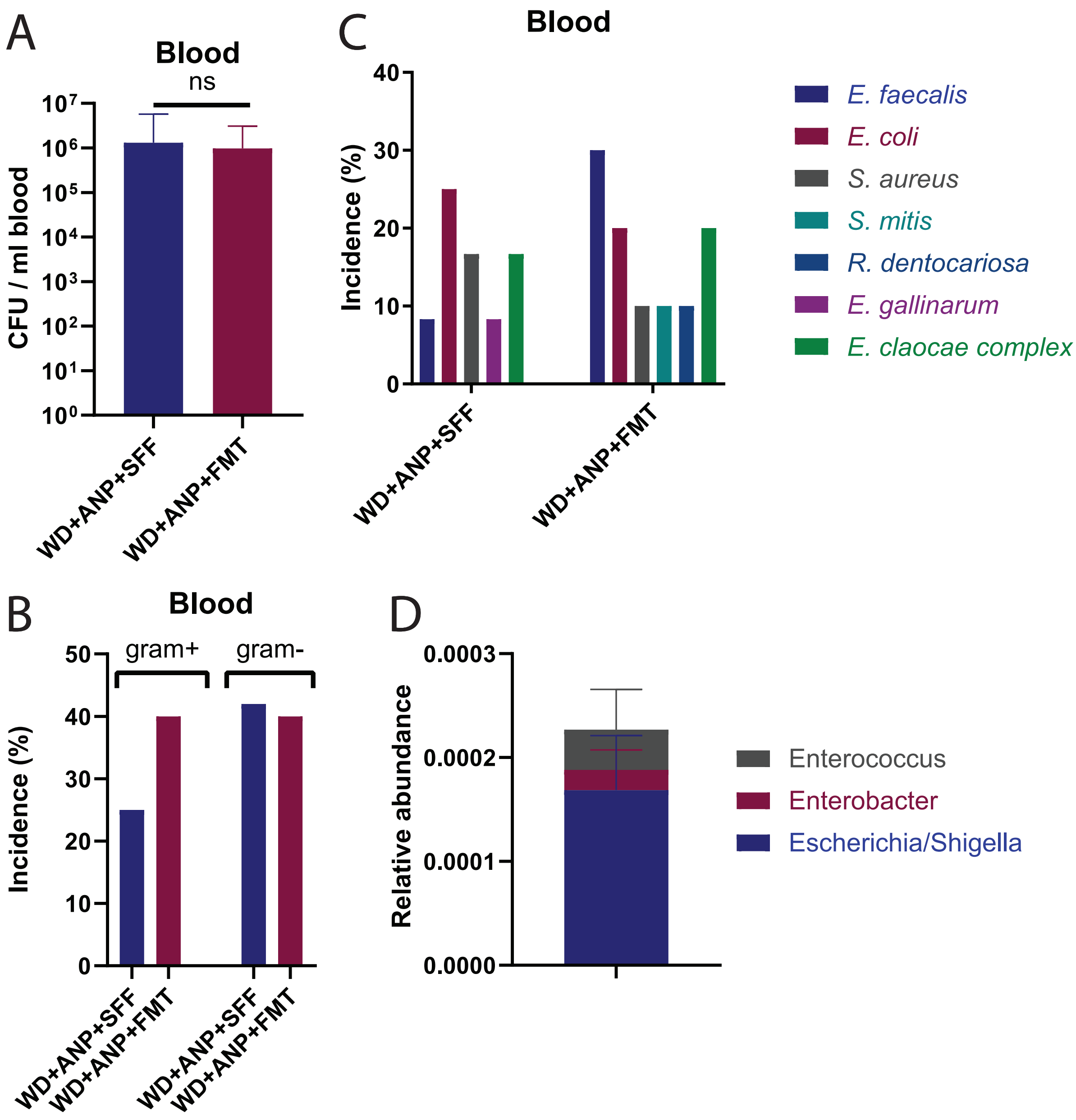

Supplementary Figure 4 (**A**) total colony forming units (CFU) of blood in FMT versus SFF group (p>0.05 by Mann-Whitney test). (**B**) Incidence of Gram(+) and Gram(-) infections of blood in FMT versus SFF group. (**C**) Incidence of cultured bacterial species in blood. (**D**) 16S rRNA gene analysis of administered FMT displaying the three most dominant genera found in pancreatic tissue. ns, not statistically significant. FMT, fecal microbiota transplantation; SFF, sterile fecal filtrate.

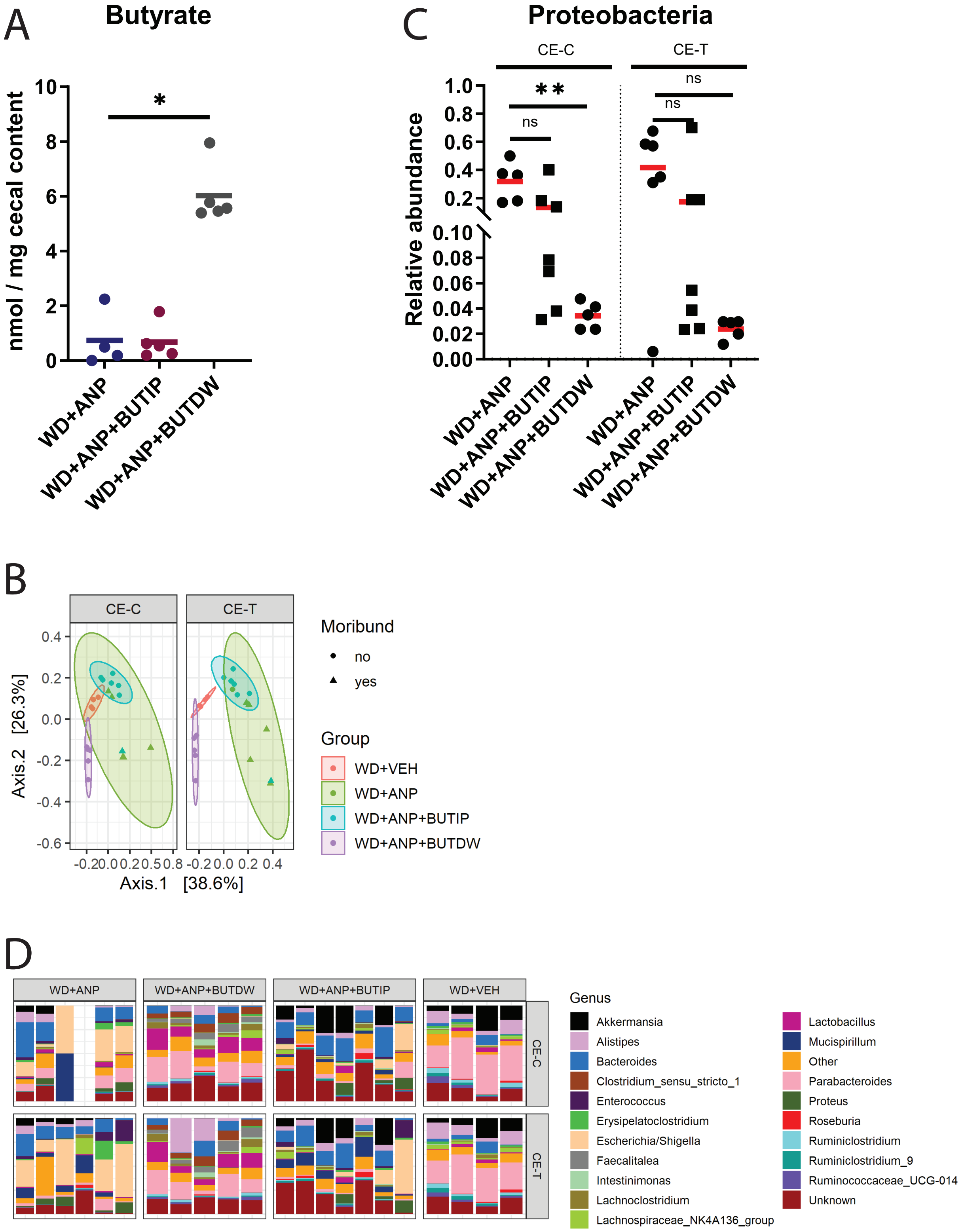

Supplementary Figure 5 (**A**) Oral butyrate supplementation partially restored cecal butyrate levels compared to untreated WD pancreatitis mice (p=0.016 by Mann-Whitney test). (**B**) 16S rRNA analysis of beta-diversity measured by weighted UniFrac indicates that BUTDW, but not BUTIP significantly influenced cecal microbiota composition (p=0.004 for WD+ANP+BUTDW vs WD+ANP, by PERMANOVA-test). Importantly, the BUTDW treatment partly restored the bacterial composition (p= for WD+VEH vs WD+ANP+BUTDW). (**C**) Relative abundance demonstrating the depletion of Proteobacteria in BUTDW group at the level of WD+VEH (p=0.008 versus WD+ANP in cecum lumen). Depletion of Proteobacteria was also observed in WD+ANP+BUTIP mice but it did not reach statistical significance. (**D**) Microbiota analysis demonstrates effect of butyrate supplementation on genus composition. *p<0.05; **p<0.01, ns, not statistically significant.

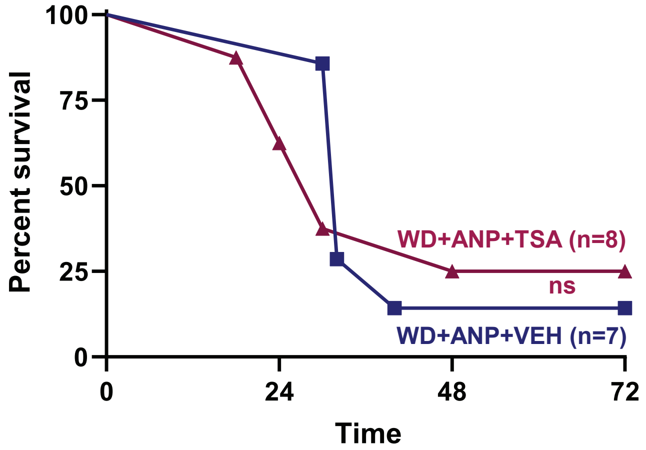

Supplementary Figure 6 Survival curves of mice treated with the histone deacetylase inhibitor trichostatin A (TSA) demonstrated no difference in survival compared to vehicle treated control group (p>0.05 by log-rank test). ns, not statistically significant.

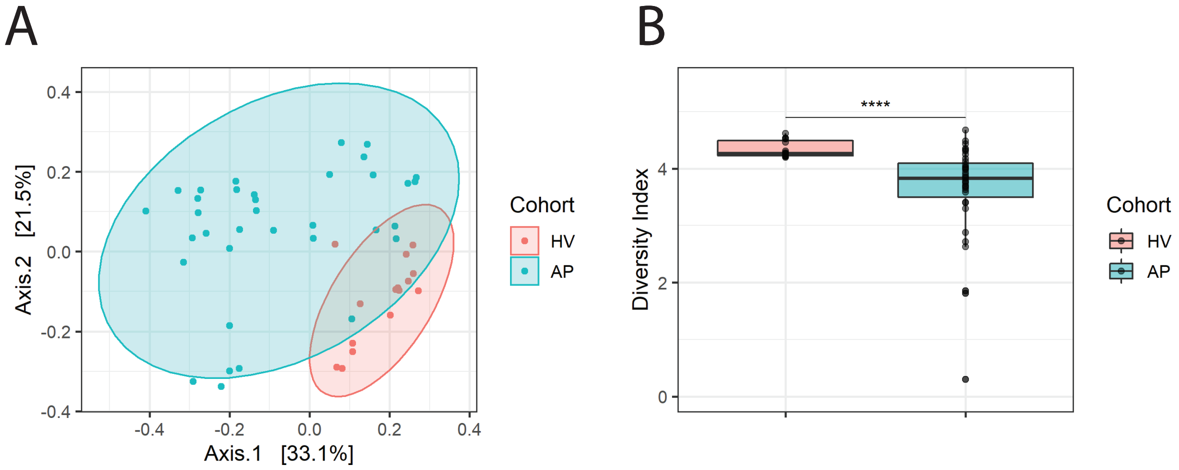

Supplementary Figure 7 (**A**) Beta-diversity of fecal microbiota of 35 patients with acute pancreatitis (AP) and 15 healthy volunteers (HV) measured by weighted UniFrac, demonstrates a significant difference in microbial composition (p=0.001 by PERMANOVA-test). (**B**) Alpha diversity of fecal microbiota measured by Shannon index, demonstrates a significant difference between AP and HV. ****p<0.0001
